# A systematic review and meta-analysis on the prevalence of vancomycin-resistant enterococci (VRE) among Nigerians

**DOI:** 10.1097/j.pbj.0000000000000125

**Published:** 2021-02-11

**Authors:** Oluwatosin Qawiyy Orababa, Jeffry Difiye Soriwei, Samuel Oluwamayowa Akinsuyi, Utibeima Udo Essiet, Olusola Michael Solesi

**Affiliations:** a Department of Microbiology, University of Lagos, Akoka Yaba, Lagos, Nigeria; b Department of Microbiology in Public Health, University of Bedfordshire, Luton, Bedfordshire, United Kingdom

**Keywords:** antibiotic resistance, *E faecalis*, *E faecium*, *Enterococcus*, Nigeria, vancomycin-resistant enterococci

## Abstract

**Background:**

Enterococci are opportunistic pathogens and are one of the most important bacteria in hospital-acquired infections. Their resistance to antibiotics such as vancomycin has led to life-threatening and difficult-to-treat nosocomial infections. The true prevalence in clinical settings in Nigeria is not well known due to the lack of a comprehensive antibiotic surveillance system. This study aims to estimate the prevalence of vancomycin-resistant enterococci (VRE) in clinical infections in Nigeria.

**Methods:**

Databases (PubMed, *African Journal Online*, and Google scholar) were searched following the Preferred Reporting Items for Systematic review and meta-analysis protocols (PRISMA-P) 2015 statements for articles reporting VRE prevalence, and were published before August 5, 2020. Data from the studies were extracted and analyzed using Microsoft Excel and Comprehensive Meta-Analysis (CMA 3.0), respectively. The pooled prevalence of VRE was estimated with the random-effects model and the 95% confidence interval (CI). The heterogeneity level was assessed using Cochran Q and *I*^2^ tests.

**Results:**

A total of 35 articles were scanned for eligibility, among which 7 were included in the study after fulfilling the eligibility criteria. The studies analyzed a total of 832 enterococci isolates and 90 VRE strains. The prevalence of *Enterococcus faecium* and *E faecalis* in this study are 361 (59.3%) and 248 (40.7%), respectively, among which 41 (63.1%) of the *E faecium* and 24 (36.9%) of the *E faecalis* were vancomycin resistant. The pooled prevalence of VRE was estimated at (95% CI; 10.0–53.9%; *I*^2^ = 93.50%; *P* < .001). The highest prevalence of VRE was reported from western Nigeria, 14.6% (95% CI; *I*^2^ = 97.27; *P* < .001).

**Conclusion:**

The prevalence of VRE in Nigeria according to the reports from this study is relatively high. The report of this study should help policymakers to put in place measures that will help curb the spread of VRE and associated resistant genes to other important clinical pathogens like *Staphylococcus aureus*.

## Introduction

The members of the genus *Enterococcus* belong to the family *Enterococcaceae* and are single/paired, catalase-negative, Gram-positive,^1^ non–spore-forming, facultatively anaerobic bacteria.^2^ They are mainly found as normal flora in the intestine of both animal and man.^3^ They are also found in the female genital tract,^4^ plants, food, and soil.^5^*Enterococcus faecium* and *E faecalis* are the most common species in this group of bacteria with *E faecalis* accounting for approximately 90% of infections caused by members of this genus.^6^ Other members of this genus that rarely cause human infections include *E mundtii*, *E casseliflavus*, *E hirae*, *E durans*, and *E raffinosus*.^7^ Members of this genus were once believed to be harmless commensals, but their roles as opportunistic pathogens have now been established.^8^ Enterococci are one of the major bacteria implicated in hospital-acquired infections such as endocarditis, neonatal sepsis, bacteremia, catheter-associated urinary tract infections (UTIs), and sometimes meningitis.^9,10^ In addition to their ability to cause infections, enterococci are well known for their antimicrobial resistance nature.

The antimicrobial-resistant ability conferred on the members of the *Enterococcus* genus through the transfer of transposons, plasmids, mutation, or chromosomal exchange^11^ makes it difficult to treat some of the infections they cause.^12,13^ This resistant gene can be transferred to other pathogens. The transfer of resistant genes from a more virulent pathogenic organism such as the members of the genus *Enterococcus* to other nonpathogenic organisms often occur in the intestines of humans and animals.^14^ Their ability to transfer antibiotic resistant genes from animal enteric bacteria to humans through the food chain has made them pathogens of global concern.^15^

Enterococci have become resistant to almost all the antimicrobial agents used against it, including vancomycin which is one of the most effective antimicrobials in the treatment of enterococcal infections.^16^ Multidrug resistance in enterococci can be associated with their inherent resistance to antibiotics, acquisition of resistance genes through mobile genetic elements, and intra- and interspecies transfer of resistance among closely related bacteria.^17^ Their biofilm-forming ability also increases their resistance to antibiotics, thus causing serious challenges in enterococal infection therapy.^18^

Vancomycin, a glycopeptide, is mainly used for the treatment of severe infections resulting from Gram-positive bacteria and acts by preventing the cross-linkage of adjacent pentapeptides, thus inhibiting cell wall formation.^19^ Resistance to vancomycin in enterococci occurs via the alteration of the peptidoglycan synthesis pathway by substituting the D-Ala-D-Ala pentapeptide terminal, in which vancomycin binds, for D-Ala-D-Lac or D-Ala-D-Ser.^20^ Vancomycin-resistant enterococci (VRE) infections are life-threatening and difficult to treat due to their resistance to varieties of clinically relevant antibiotics.^21^ VRE are transmitted in clinical settings through inanimate surfaces such as thermometer, gloves, bed rails, stethoscopes, cutleries, and healthcare workers.^22^*E faecium* is a member of the ESKAPE (*E faecium*, *Staphylococcus aureus*, *Klebsiella* spp, *Acinetobacter baumannii*, *Pseudomonas aeruginosa*, *Enterobacter* spp) pathogens, which are high-risk antibiotic-resistant pathogens.^23^*E faecium* is notable among them due to its ability to intrinsically resist different antibiotics including beta-lactams and aminoglycosides.^24^

The true prevalence of VRE in clinical settings, same as other multidrug resistant (MDR) pathogens, is not well known in Nigeria. This can be linked to the lack of an effective national MDR surveillance system in the country. Although there have been few studies reporting VRE prevalence in different parts of Nigeria, there has not been a systematic review and meta-analysis that exclusively analyzed the pooled prevalence of clinical isolates of VRE in Nigeria as at the time this study was carried out. This study was performed to analyze the prevalence, and distribution of VRE strains in Nigeria by summarizing the findings of previous cross-sectional studies carried out in different parts of the country.

## Methods

This systematic review was conducted following the guidelines provided in the Preferred Reporting Items for Systematic review and meta-analysis protocols 2015 statements and guidelines.^25^

### Search strategy and sources of information

Databases such as PubMed, *African Journal Online*, EMBASE, and Google Scholar were searched for articles published before August 5, 2020. The reference lists of relevant articles were also used to obtain supplementary articles to be included in this study. The search included a combination of the following words and their synonyms; “*Enterococcus*,” “antibiogram,” “vancomycin resistance,” and “Nigeria”. The databases were searched independently by three reviewers (O.Q.O., U.U.E. and J.D.S.). The last search date was August 28, 2020.

### Eligibility criteria

#### Inclusion and exclusion criteria

All cross-sectional studies that reported the prevalence and vancomycin resistance in enterococci from clinical specimens in Nigeria were included in this study for further analysis. Studies that reported the prevalence and antibiotic resistance of enterococci from sources other than humans were excluded from this study. Studies with inaccessible full texts, no reports of vancomycin resistance, and/or with no specified total number of enterococci isolates were also excluded. Lastly, case reports and review studies were excluded from this analysis.

### Quality assessment

The quality of the studies included in this review was assessed independently by 2 reviewers using the modified Critical Appraisal Checklist for prevalence studies recommended by the Joanna Briggs Institute, which contains 9 questions that were addressed for each of the eligible studies by the reviewers.^26^

#### Data extraction

Data from eligible studies were extracted independently by 2 reviewers (J.D.S. and S.O.A.) and checked by a third reviewer (O.Q.O.). Disagreements among the reviewers were resolved through discussion. The following data were extracted from included studies; first author and publication year, study design, country region, study subject, sample size, enterococci prevalence, species isolated, antibiotic susceptibility testing method, and prevalence of VRE.

#### Data analysis

Subgroup prevalence was analyzed by considering region, antibiotic susceptibility testing method, and specimen type analyzed. The random-effects model was used to determine VRE pooled prevalence in this analysis because of the acknowledgment of heterogeneity in cross-sectional studies carried out in diverse environments. The Cochrane Q-test and the inverse variance index (*I*^2^) were used to evaluate the heterogeneity in this study.^27^ Comprehensive Meta-Analysis (CMA) version 3.0 for windows was used to analyze the data. The statistical analysis was done by O.Q.O.

## Results

**Figure 1:**
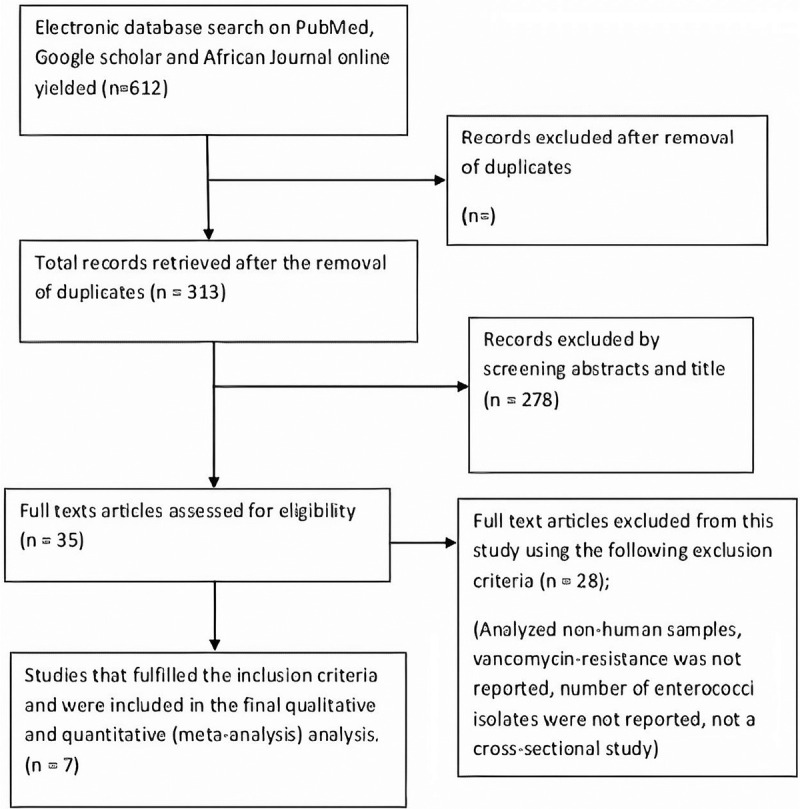
Flow chart of the study selection procedure.

A total of 35 articles were identified through searching databases such as PubMed, *African Journal Online*, and Google Scholar. After full text screening for eligible articles, only 5 articles were added to this review. Thirty of the articles were not eligible for this review. Two^2^ additional articles were obtained through searching of reference lists. Finally, 7 articles were included in this study. Figure 1 shows the procedure for study selection.

### Characteristics of the included studies

**Figure 2:**
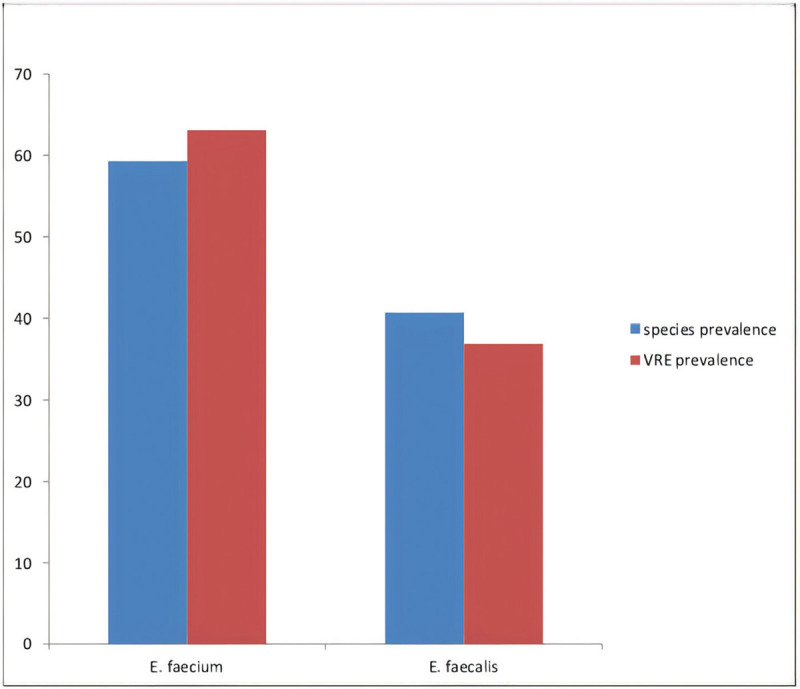
Vancomycin-resistant enterococci (VRE) prevalence among clinical *E faecium* and *E faecalis* isolates in Nigeria.

Only cross-sectional studies were analyzed in this review. A total of 7 studies were analyzed in this review.^4,12,28–32^ Most of the studies included were reported from the western (n = 3)^11,20,24^ and northern (n = 3)^4,29,31^ regions of Nigeria. Only one of the studies was from the eastern region of Nigeria.^30^ This review analyzed a total of 832 enterococci isolates from 2760 clinical samples tested, among which 90 were VRE strains. *E faecalis* and *E faecium* were the most isolated enterococci species. Three^3^ of the studies isolated a total of 177 enterococci from multiple clinical specimens,^29,30,32^ 2 studies isolated 638 from stool samples^12,31^ whereas 1 study each isolated 8 and 13 enterococci from urine^4^ and rectal swabs,^28^ respectively. Five^5^ of the studies used the disc diffusion method,^4,12,29,30,32^ whereas 2 used dilution/minimum inhibitory concentration method^28,31^ to determine VRE. The prevalence of VRE from the studies ranged from 0.01% in the North-east to 34% in the North-central. The prevalence of *E faecium* and *E faecalis* from these studies are 361 (59.3%) and 248 (40.7%), respectively, among which 41 (63.1%) of the *E faecium* and 24 (36.9%) of the *E faecalis* were resistant to vancomycin as shown in Figure 2.

### Studies that included vancomycin-resistant enterococci isolates from Northern Nigeria

Northern Nigeria comprises 3 zones, North-west, North-east, and North-central. Three^3^ studies that reported the prevalence of VRE in Northern Nigeria were included in this study.^4,29,31^ The first study reported 8 enterococci isolates from urine samples in Amino- Kano Teaching Hospital in the North-western part of Nigeria. The prevalence of VRE was 4 (50%). *E faecalis* was the only species of enterococci isolated.^4^ The second study was conducted in the North-central and involved 102 enterococci isolates obtained from multiple clinical specimens (stool, urine, wound, and swabs). The prevalence of VRE in the study was 34 (33.33%).^29^ The third study obtained 561 isolates from stool samples and reported a VRE prevalence of 0.01%.^31^

### Studies that included vancomycin-resistant enterococci isolates collected in western Nigeria

Three^3^ studies were reported from western Nigeria (South-west).^12,28,32^ The first study screened 100 stool samples, reported 73 (73%) enterococci isolates identified as and a VRE prevalence of 9 (13.80%) among the identified *E faecalis* and *E faecium* isolates.^12^ The second study isolated 13 (4.07%) from 319 rectal swabs with a VRE prevalence of 13 (4.07%).^28^ The third study analyzed 118 multiple clinical specimens (blood, urine, wound swabs, sputum, and stool), isolated 7 (5.9%), and 3 (42.9%) VRE strains.^32^

### Studies that included vancomycin-resistant enterococci isolates collected in eastern Nigeria

Only 1 study from the eastern region was included in this study. A total of 1048 clinical specimen were collected in the study, and 68 (6.49%) enterococci were isolated. The prevalence of VRE in the study was determined to be 21 (30.9%).^30^

### The pooled prevalence of vancomycin-resistant enterococci

**Figure 3:**
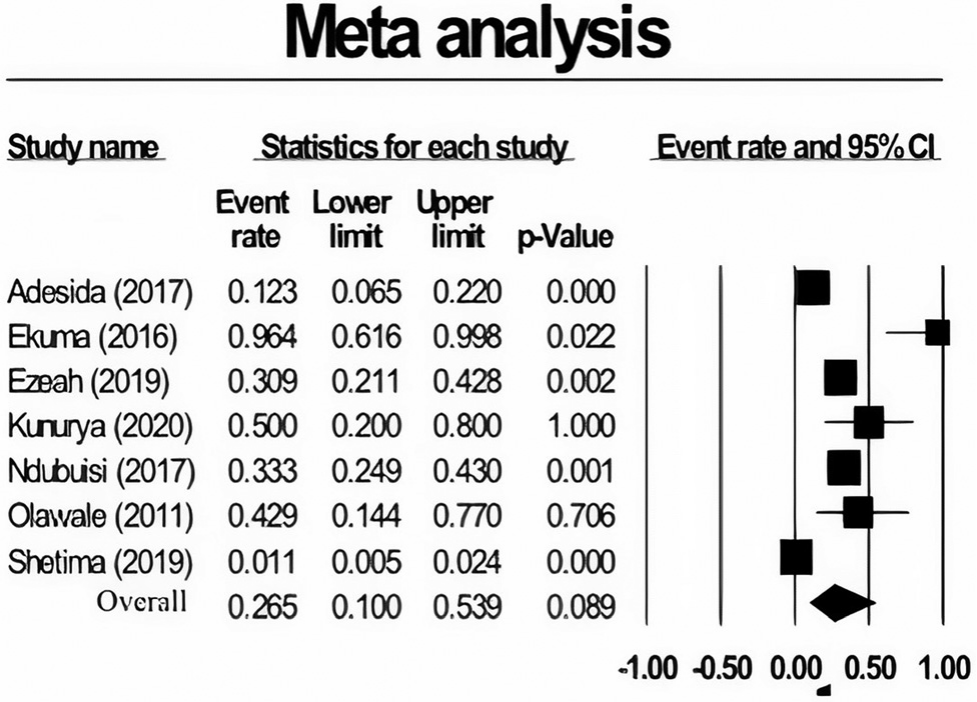
Forest plot showing the pooled prevalence of vancomycin-resistant enterococci (VRE) among Nigerians. CI = confidence interval.

The pooled prevalence VRE in this study was estimated at 26.5% (95% confidence interval [CI]; 10.0–53.9; *I*^2^ = 93.50%; *P* < .001) (Fig. 3). There was significant heterogeneity (*Q* = 92.32%; *I*^2^ = 93.50%; *P* < .001). The presence of publication bias was analyzed using the funnel plot (Fig. 2). VRE prevalence based on region gave estimates of 49.6% (95% CI; 8.3–91.5; *I*^2^ = 86.87%; *P* < .001) for the western region, 14.6% (95% CI; *I*^2^ = 97.27; *P* < .001) for the northern region and 30.9% (95% CI; 1.1–72.9) for the eastern region (Table 1). Subgroup analysis on the sample showed a high estimated prevalence of VRE from urine samples (50.0%), and rectal swab (96.4%). Multiple specimen sites also increased the chances of isolating VRE. Also, the pooled prevalence of VRE using the disc diffusion method was 29.1%, whereas it was 32.4% using dilution/minimum inhibitory concentration (Table 2).

**Table 1:** Characteristics of eligible studies

First author (publication year)	Study design	Study region	Sample size	Specimen type	Enterococci prevalence, N (%)	*Enterococcus* species isolated	AST method	Prevalence of VRE (%)
Adesida (2017)	CS	South-west, Western region	100	Stool	73 (73.0)	*E feacalis* *E faecium*	Disk diffusion	9 (13.80)
Ekuma (2016)	CS	South-west, Western region	319	Rectal swabs	13 (4.07)	*E faecium*, *E gallinarum*, *E casseliflavus*	Dilution/MIC, PCR	13 (4.07)
Ezeah (2019)	CS	South-east, Eastern region	1048	Urine, sputum, stool, aspirates, CSF, high vaginal swab, urethral, wound Nasa, ear and anal	68 (6.49)	*E faecalis* and *E avium*	Disk diffusion Method	21 (30.90)
Kunurya (2020)	CS	North-west, Northern region	114	Urine	8 (7.00)	*E faecalis*	Kirby Bauer Disk diffusion Method, and MIC	4 (50.00)
Ndubuisi (2017)	CS	North-central, Northern region	500	Stool, urine, wound swab, Environmental samples	102 (24.00)	*E faecalis* *E faecium,* *E Casseliflavus,* *E mundtii,* *E dispar,* *E shirae,* *E avium* *E gallinarum*	Kirby Bauer Disk diffusion Method	34 (33.33)
Olawale (2011)	CS	South-west, Western region	118	Blood, urine, wound swabs, sputum, and stool	7 (5.90)	*E faecalis* *E faecium*	Disk diffusion	3 (42.90)
Shettima (2019)	CS	North-east, Northern region	561	stool	561 (100%)	*E faecalis*, *E faecium*, *E casseliflavus*, *E hirae*, *E durans*, *E mundtii*, *E raffinosus, E dispar*	Dilution/MIC	6 (0.01)

AST = antibiotic susceptibility testing; VRE = vancomycin-resistant enterococci.

**Table 2:** Pooled prevalence of vancomycin-resistant enterococci according to subgroups

Subgroups	Number of studies	Number of enterococci tested	Pooled prevalence of VRE, N (%)	95% Confidence interval (CI)	*I*^2^	*P*
Region						
Western	3	93	25 (49.6)	8.3–91.5	86.87	<.001
Eastern	1	68	21 (30.9)	21.1–42.8	–	–
Northern	3	671	44 (14.6)	1.1–72.9	97.27	<.001
AST method						
Disc diffusion	5	258	71 (29.1)	18.8–42.2	67.35	.016
Dilution/MIC	2	574	19 (32.4)	0.0–99.9	96.34	<.001
Specimen type						
Stool	2	634	15 (3.8)	0.3–32.7	95.52	<.001
Urine	1	8	4 (50.0)	20.0–80.0	–	–
Rectal swab	1	13	13 (96.4)	61.6–99.8	–	–
Multiple sites	3	177	58 (32.8)	26.3–40.1	–	.801

AST = antibiotic susceptibility testing; MIC = minimum inhibitory concentration; VRE = vancomycin-resistant enterococci.

## Discussion

Enterococci were initially regarded as a harmless group of bacteria, but are now among the most frequent bacteria in hospital-acquired infections, just behind *Escherichia coli* and *Staphylococcus*.^33^ Their ability to intrinsically resist various antibiotics used in clinical settings has made them a global public health threat. Infections from VRE have been reported from different studies around the world.^34–36^ The lack of an antimicrobial surveillance system in Nigeria has made it difficult to determine the true burden of VRE all over Nigeria. Many cross-sectional studies have been conducted to determine the burden of VRE in different parts of Nigeria, but there has not been a comprehensive review covering different parts of Nigeria. This study was carried out to analyze the prevalence of VRE isolates in Nigeria.

A total of seven^7^ studies reporting the prevalence of VRE from different parts of Nigeria were included in this systematic review and meta-analysis. This study showed that the most prevalent *Enterococcus* species in clinical infections are *E feacalis* and *E faecium*. These 2 species of *Enterococcus* are the most common enterococcal representatives found in human intestines and are also implicated in majority of human enterococcal infections.^37^*E faecalis* and *E faecium* have been considered as the third and fourth most common nosocomial pathogens globally by the European Centre for Disease Prevention and Control.^38^ This study observed a prevalence of 59.3% and 40.7% in *E faecium* and *E faecalis,* respectively. This conforms to a report from a study from the United States that reported a higher prevalence of *E faecium* from clinical settings.^39^ Many studies around the world have, however, reported the predominance of *E faecalis* in clinical infections.^40–42^ Differences in the geographical region can be a major factor in this variation. Although *E faecalis* has been known to be the most common *Enterococcus* sp in clinical infections, infections due to *E faecium* have recently shown a significant increase.^43^ An increase in *E faecium* prevalence in clinical infections have been linked to increase in the use of medical devices and increased duration of carriage.^39^

A higher prevalence of VRE was observed in *E faecium* (63.1%) compared to *E faecalis* (36.9%) in this study. Other studies have also reported a similar pattern of VRE prevalence at the species level.^44^*E faecium* is known to easily acquire resistant genes from its environment compared to *E faecalis*, this has been a major factor in its multidrug resistance nature.^45^ Vancomycin-resistant *E faecium* is classified as a priority 2 pathogen with significant threat to public health according to the World Health Organization.^46^*E faecium* has been implicated in serious enterococci infections with limited therapeutic options.^47^ Resistance to vancomycin in enterococci is mediated by the 9 different van operons.^19^ Eight of these operons (*vanA*, *vanB*, *vanD*, *vanE*, *vanG*, *vanL*, *vanM*, and *vanN*) mediate acquired vancomycin resistance, whereas 1 mediates (*vanC*) inherent vancomycin resistance.^48,49^ Among these operons, *vanA* and *vanB* are the most common in VRE isolates of human origin.^50^

The pooled prevalence of VRE from clinical settings in this study is 26.5%. This report is similar to what was reported in Asia (24%) and North America (21%).^51^ Conversely, this prevalence is relatively high compared to reports from Ethiopia (14.8%),^52^ Iran (14%),^40^ England (12.2%),^53^ and South Korea (16%).^54^ The variations observed among these studies can be associated with the study population, geographical region, and sample size. The high prevalence of VRE in Nigeria could be as a result of antibiotic misuse among Nigerians. Antibiotics are easily available over the counter in many parts of Nigeria, which has led to their use without proper diagnosis or prescription from qualified health personnel. VRE are usually present as normal flora in the intestines of animals and humans without causing infections.^55^ However, they may colonize and disseminate when anti-anaerobic antibiotics, including vancomycin, that displaces susceptible enterococci are used, thereby providing a suitable condition of VRE to invade.^56^ Antimicrobial stewardship programs should be established in hospitals in Nigeria to control the use of antibiotics in the treatment of infections. The presence of VRE in healthy humans was affirmed in one of the studies analyzed in this review as VRE isolates were reported in healthy humans.^12^ VRE infections are rapidly increasing in hospitals globally due to their ability to survive on inanimate surfaces such as beds, ventilation systems, benches, and implanted surgical devices for longer periods.^57^ Mortality rate as high as 63% has been associated with VRE infections.^58^ Risk factors such as immunosuppression,^59^ comorbid illness,^58^ and exposure to antibiotics^60^ are factors that promote the establishment of VRE infections.

The prevalence of VRE according to regions showed that the western part of Nigeria has the highest prevalence of 49.6%; this is more than three times the prevalence in the northern region (14.6%), which had the lowest. The variation in this prevalence could be as a result of the differences in a population study, weather condition, and activities of the populace from the regions. There was no eligible study from some parts of the southern region (South-South) of the country while there were few studies from other regions, more studies that conform with international practices are required from different parts of Nigeria to get a better picture of the distribution of VRE in Nigeria. VRE prevalence based on clinical specimen was high for rectal swab (96.4%) and urine (50%) compared to other samples. This is not surprising as enterococci are the third-highest common organisms in UTIs, and they also form part of the microflora in the gut of humans.^61^ Studies that analyzed multiple clinical specimen isolated more VRE strains than studies that analyzed just one type of clinical specimen. The presence of enterococci in different clinical specimen can be attributed to their presence in the gut as normal flora and their ability to cause varieties of infections ranging from UTI to bacteremia to endocarditis.^9^

## Conclusion

Drug-resistant nosocomial pathogens such as VRE are a great menace to both patients and health-care workers as they increase hospital stay, cost of infection treatment and sometimes lead to death. This study has shown that there is high prevalence of VRE infections in Nigeria, which might increase if measures are not put in place to reduce it. There is urgent need for a national VRE surveillance in hospitals all over the country to determine the true risks posed by these drug-resistant pathogens and to aid the development of policies that will reduce the spread of MDR in Nigeria. There is also a need for more prevalence studies all over the country especially in regions where they are deficient. Lastly, antimicrobial stewardship programs should be implemented in hospitals throughout the country to monitor the use of antibiotics and reduce selective pressure of antibiotics on important clinical pathogens in hospital environments.

## Acknowledgements

None.

## Conflicts of interest

The authors declare no competing interests.
